# Exploring Factors Associated with Diabetic Retinopathy Treatment Compliance Behaviour in Cape Town, South Africa

**DOI:** 10.3390/ijerph182212209

**Published:** 2021-11-20

**Authors:** Annalie Wentzel, Zandile June-Rose Mchiza

**Affiliations:** 1 School of Public Health, University of the Western Cape, Bellville, Cape Town 7535, South Africa; jmchiza@uwc.ac.za; 2 Non-Communicable Disease Research Unit, South African Medical Research Council, Parow Valley, Cape Town 7501, South Africa

**Keywords:** diabetes mellitus, diabetic retinopathy, compliance behaviour, retinal screening, SARS-CoV-2, Cape Town

## Abstract

Complete patient adherence to treatment for diabetic retinopathy (DR) is critical to limit vision loss. There is a dearth of evidence regarding the reasons why South African patients referred for suspected vision-threatening DR stay compliant to or default their treatment. The current study sought to explore factors associated with treatment compliance among patients living with diabetes who have been referred for suspected vision-threatening DR in the Northern/Tygerberg sub-Structure (NTSS) public health care system of Cape Town, South Africa. A qualitative research approach was used where semi-structured in-depth interviews were conducted with 13 adult patients living with DR, and 2 key informants who are primary eye care providers. Thematic data analysis was conducted using taguette.org. Fear of going blind was the most notable patient-related factor associated with compliance. Notable patient-related barriers reported were forgetfulness and a poor state of health. Notable institution-related barriers included suboptimal information received from health care service providers, poor referral management by the organisation delivering retinal screening services, as well as the inaccessibility of the main NTSS hospital via telephone calls. All these factors were confirmed by the key informants of the current study. Finally, all patients and key informants agreed that SARS-CoV-2 negatively affected patients’ adherence to their DR treatment. Hence, scaling up of health care, referral, and appointment setting services could increase the uptake of treatment and retinal screenings among patients attending the Cape Town, NTSS public health care system.

## 1. Introduction

Diabetic retinopathy (DR) is a vision-threatening complication of diabetes mellitus [1] and a leading global cause of vision loss [2]. There is substantiated international evidence that suggests DR treatment non-compliance rates are unacceptably high [3,4,5,6]. Similarly, in South Africa’s public health system, sight-threatening DR prevalence rates of 11.0% and 7.5%, respectively, have been found [7,8]. Cockburn et al. [9] further indicated that DR is a critical cause of preventable visual impairment in Cape Town, causing 8%, 11%, and 2% of blindness, severe visual impairment, and partial visual impairment, respectively, in persons 50 years and older.

The risk of vision loss from DR can be mitigated by early detection, referral for the needed health care, and ophthalmological treatment, all in combination with strict glucose control [9]. Given the high burden associated with partial (i.e., a need for expensive visual aids [10]) or total vision loss (i.e., the loss of income [11]) and poor quality of life [12] in South Africa, strict DR management compliance is needed. While there is a lack of data regarding the extent and factors associated with DR compliance in the South African context, international studies attribute compliance to the patient-related, service provider/institution-related, and treatment-related factors [3,13].

According to the Social Determinants of Health Model by Whitehead and Dahlgren [14], all the aforementioned factors could be categorised into immediate (individual-related), underlying (community- and societal-related), and basic (socioeconomic- and environmental-related) levels of the public health care system. For instance, when Whitehead and Dahlgren’s [14] conceptual model is adopted and applied to the current research, we may be able to associate patient DR compliance with immediate factors such as age, gender, health status, financial trade-offs, health beliefs, the fear of medical procedures, and forgetfulness. Then, when it comes to the community and societal factors, we can associate patient DR compliance with underlying determinants such as familial and societal support, as well as education and services offered by health care providers. These factors also interrelate with the aforementioned immediate factors. Finally, we may assume that patient DR compliance also associates with the “so-called” basic factors such as socioeconomic and environmental circumstances. These factors also interrelate with both immediate and underlying factors. Into the entire mix of interrelated factors, it is important to consider the accessibility of DR health care services in the wake of the SARS-CoV-2 epidemic. There is growing global evidence that suggests that the management of non-communicable diseases (NCDs)—chronic, non-infectious diseases caused by genetic, physiological, environmental, and behavioural factors such as diabetes—has been interrupted or harmed by the emergence of the SARS-CoV-2 pandemic [15,16,17]. However, the mechanism by which this pandemic is impacting NCD treatment, including DR management compliance in South African patients, is currently unknown.

Anecdotal evidence and personal communication (Ziskind, A. Tygerberg Hospital Ophthalmology Department. Personal communication, 2018, September) with a DR expert in the Northern/Tygerberg sub-Structure (NTSS) public health care system of Cape Town suggested that an unacceptable number of patients in this system default from their ophthalmological DR treatment. Hence, research such as the current is necessary if we are to bridge the aforementioned research gap. The current research, therefore, explored factors associated with DR treatment compliance among the patients living with diabetes, who have been referred for suspected vision-threatening DR in this aforementioned public health care system of Cape Town. The awareness and understanding of these contextual factors could lead to improved interventions directed at limiting preventable vision loss, especially in underserved communities of Cape Town and South Africa.

## 2. Materials and Methods

### 2.1. Study Design

We used a qualitative research design to explore factors related to compliance behaviour among patients living with DR. This design enabled us to explore factors unique to patients in our target setting, as there was a dearth of data available on the DR topic, especially in lower-income/underserved Cape Town communities [3,9,18]. This flexible qualitative research method also allowed us to detect new information rather than the data outlined in the international literature we reviewed.

Compliance can be defined as “the extent to which the patient’s behaviour matches the prescriber’s recommendations” [19]. For this research, DR treatment compliance refers to a patient being present for all ophthalmological consultations or treatments as scheduled by the treating ophthalmologist. Diabetic retinopathy management compliance refers to a patient attending a periodic retinal screening as advised by a health professional and attending general diabetes-related appointments at their clinic.

### 2.2. Research Setting

In the current research, we sampled patients from various government-subsidised primary health care facilities in the NTSS public health care system of Cape Town, South Africa. These primary health care facilities—or “day hospitals”, as they are colloquially called—generally cater to people without medical aids and those who cannot afford private health care. There are sizable income disparities within different Cape Town communities, with a high incidence of relative poverty (45.9%), largely as a result of historical oppression and poor governing [20,21,22,23]. Cape Town had a Gini coefficient of 0.62 in 2018, indicating immense inequality [22].

The NTSS had an estimated 1.08 million inhabitants, the majority of whom (>80%) were receiving medical care primarily from their community day hospitals [20,23]. In 2019, 45.9% of the Cape Town population were living below the upper poverty threshold of 1227 ZAR (83.2 USD) per person per month [22]. The official and expanded unemployment rates for January–March 2021 in Cape Town (with data for this survey collected closer to the current research fieldwork) were 22.5% and 32.2%, respectively [24]. In 2019, less than half of the Cape Town population (48.1%) had completed high school, and nearly one-fifth of households (17.6%) were living in informal dwellings [24]. Moreover, most recent studies have placed the diabetes prevalence rate for the Western Cape Province between 21.2% and 23.8% [25,26]. These prevalence rates are significantly higher than the national diabetes prevalence rate of 12.7% [27].

### 2.3. Study Population

The current study population comprised 19 years and older people living with diabetes who were attending day hospitals in the NTSS for medical care, who underwent a retinal screening (a test to detect DR) at their NTSS primary care facility, and who were referred to the main local hospital’s ophthalmology department for DR treatment. This population contained eligible patients with attributes and experiences crucial for our research questions [24]. Key informants were included and consisted of optometrists with a special interest in DR, who perform retinal screenings within our target setting.

### 2.4. Study Sample

Thirteen patients and two key informants were sampled, using purposive sampling to generate a variety of rich data regarding DR treatment compliance behaviour within our target setting [28,29]. The specific attributes identified to ensure rich data included (i) diabetes mellitus diagnosis; (ii) retinal screening attendance at an NTSS day hospital, 18 months before sampling for the current research; (iii) a referral to the main local hospital for treatment for the suspected vision-threatening DR, 18 months before sampling for the current research [29,30]. It is important to make the distinction that we sampled patients that underwent retinal screenings at their NTSS primary health care facility, as these were the only data we had access to. The data indicated whether a patient had suspected vision-threatening DR and whether they were referred to the main NTSS hospital for treatment. In this case, vision-threatening DR—as identified by optometrists responsible for grading the retinal images and referring patients—were classified as proliferative and severe non-proliferative DR, as well as DR in which hard exudates or microaneurysms were present within 1 disc diameter of the fovea centralis (in one or both eyes). There were no follow-up data available from the primary eye care organisation for any of the patients that were sampled at the time of data collection, which we can partly attribute to suspended services resulting from the COVID-19-related lockdown. Furthermore, we did not have access to patient data from the main NTSS hospital that is responsible for examining and treating patients with DR. Therefore, we could not confirm at which stages of DR the patients were, what the disease prognoses were, or which treatments, if any, were administered by ophthalmologists at the main NTSS hospital. However, the expected DR treatment plan would usually be administered over multiple outpatient-based visits and would likely consist of laser photocoagulation and/or intravitreal anti-vascular endothelial growth factor (anti-VEGF) injections. A pars plana vitrectomy would be considered in more severe proliferative cases [31].

The two key informants selected were optometrists—with a special interest in DR—working within the target setting; they were also familiar with our sample population. As such, they had valuable knowledge about the study topic within the study setting due to their proximity and experience [32].

We accessed prospective patients’ contact details via the organisation performing primary eye care services (including retinal screenings) in the target setting. We only included patients conversant in Afrikaans or English as these are the two main languages predominantly spoken by the patients attending NTSS health facilities. This also allowed the primary investigator to take charge in completing the interviews independently, as she is fluent in Afrikaans and English. Only patients who were 19 years or older were included in the current research.

The total sample size of 15 was justifiable due to the reasonably homogenous sample population and qualitative study design [28]. Moreover, data saturation for thematic analysis can occur after 12 patient interviews [33]. After all, the researchers of the current study were interested in gaining in-depth information regarding patients’ experiences and their complex social realities, rather than generalising the outcomes of this research to the broader South African population [34].

### 2.5. Data Collection

Individual, semi-structured, in-depth interviews took place from mid-August 2020 until early September 2020. The SARS-CoV-2 lockdown in South Africa at that time, paired with the high-risk status of diabetic persons, resulted in the current research being conducted via telephone interviews as opposed to the initially planned in-person interviews. This was done to minimise the risk of SARS-CoV-2 transmission in every way possible. We sent electronic information sheets and delivered the details of the research telephonically to the patients. Telephone communications also allowed patients to ask any questions they may have. Thereafter, we sent consent forms electronically to ask patients to give written informed consent before conducting interviews. We also asked patients personal identifying questions before pressing the button to record the conversations. Patient and key informant data remained confidential. To minimise respondent fatigue, interviews did not exceed 40 min [29].

Interviews explored patient knowledge and experiences relating to diabetes and DR management, particularly barriers and motivating factors in attending appointments for ophthalmological DR treatment. Key informant interviews explored perceptions relating to patient compliance behaviour, both to expand collected data and to triangulate with patient data [35]. We used pre-tested semi-structured interview guides for the patient and key informant interviews, respectively (Supplementary Materials Text S1: Semi-structured interview guide—patients; Supplementary Materials Text S2: Semi-structured interview guide—key informants). The flexibility of semi-structured interviews allowed us to discover new information and explore diverse perceptions while facilitating comparisons and simplifying the data analysis [36]. Interview questions were informed by data discovered during the preliminary literature review.

The primary researcher conducted all interviews. Being an optometrist with prolonged exposure to the target setting, this researcher had insights into patients’ social contexts and could easily and successfully probe for extensive data during interviews. The investigator fed post-interview notes and perceptions into a research journal to monitor how her perceptions affect data analysis and to improve reflexivity [29].

The primary researcher transcribed audio data verbatim, then translated all Afrikaans interviews to English in such a way as to reflect nuances and maintain the authenticity of the data. The transcripts and audio data were sent to a qualified independent party to verify the accuracy. In addition, this was double-checked by the supervisor of the primary investigator who is also an expert in diabetes-related research.

### 2.6. Data Analysis

The transcripts were analysed by the primary researcher using thematic analysis. Taguette.org, a free web-based qualitative tool was used to study the data, search for interesting or recurring patterns, and code the data accordingly [29]. All rationale were recorded into a research journal during the coding stage for reflexivity [29]. All the developed codes were sent to the research supervisor to be reviewed independently. After coding, codes were assembled into categories and sub-themes that reflected how socioeconomic and environmental, community and societal, individual, and SARS-CoV-2 factors affect DR treatment compliance behaviour [37]. Potential themes were then assembled and refined for coherence and accurate data reflection by both the primary researcher and the supervisor [38].

### 2.7. Ethics Considerations

Initial ethics approval for in-person interviews was obtained from the Biomedical Research Ethics Committee of the University of the Western Cape on 5 March 2020 (Reference No: BM20/1/8). Due to the COVID-19 lockdown, an amendment to the protocol was undertaken to motivate the use of telephone calls for interviews. The request was granted by the same committee (Reference No: BM20/1/8). Permission to access patient files to recruit participants was sought from the organisation conducting retinal screenings within the NTSS. Further permission to conduct the current clinical research in the NTSS was granted by the Western Cape (Provincial) Health Research Council and the respective NTSS primary health care facilities. The research also adhered to the guidelines outlined in the South African Department of Health Ethics in Health Research Principles [39].

## 3. Results

### 3.1. General Description

We interviewed 13 patients who had been referred for treatment of vision-threatening DR, and two key informants working in the target setting. One of these patients, an 87-year-old female, elected to have her full-time care-worker (noted as P1 carer) who lives with her and attends all appointments with her to be part of the interview due to the patient’s ill health. Selected demographic information for the 13 patients can be found in Table 1.

In Table 1, it is outlined that the mean age of the patients was 56.4 years, with 61.5% female and 38.5% male patients.

The majority of the patients (84.3%) had type 2 diabetes, with the majority of these patients (84.3%) diagnosed more than 4 years before the current research.

The best-corrected (with spectacle lenses) visual acuity for patients, which was measured on the day of the retinal screening, are given in Table 1. The majority of patients (69.2%) had no binocular visual impairment (6/6 ≤ VA < 6/12), 15.4% had mild visual impairment (6/12 ≤ VA ≤ 6/18), and 7.7% were blind (VA < 3/60) [40].

Patient compliance with prescribed DR treatment is outlined in Table 2. Overall, 61.5% of patients were compliant with their treatment, while 7.7% were partially compliant, and 30.8% were not compliant.

### 3.2. Basic Factors: Socioeconomic- and Environment-Related

Patients reported multiple socioeconomic and environmental factors to affect their compliance behaviour (Table 3). On probing patients about the access of transport to attend DR management appointments, this was found to not affect the patients’ DR compliance behaviour. The majority of patients cited that community day hospitals (where retinal screenings take place) were within their walking distance. Patients who reported living far from the day and main NTSS hospitals suggested that they relied on family, friends, community members, or “public transport” to reach the relevant health care facilities. However, securing an appointment for either a retinal screening or ophthalmological treatment proved more difficult. Difficulty in securing retinal screening appointments, before and after the SARS-CoV-2 outbreak, impeded patients’ access to preventative eye care. Moreover, errors in the referral pathway from the retinal screening at community day hospitals to the main NTSS hospital resulted in two patients reporting missing out on their DR treatment appointments. Key informants were unaware of any errors in the referral system. In the current research, it was also highlighted that patients struggled to contact the main NTSS hospital where treatment could be sought telephonically. This resulted in patients having difficulty confirming their appointment dates.

The quality of services that patients experienced at community day hospitals for retinal screenings varied. The main complaints were long waiting times, rushed tests, and limited information offered. The sentiments were reflected by key informant 2, who stated that they often give minimalistic explanations due to time constraints, limited resources, and being over-burdened with the number of patients. Only one patient cited service quality as a reason for defaulting on retinal screenings, although he stated that he intends to get private medical care once he can afford it. Presumably, the other patients cannot afford private care and therefore do not have a choice but to attend services at day hospitals.

Overall, it seemed as though patients were satisfied with the care they received at the main NTSS hospital’s ophthalmology department, despite long waiting times. Patients also praised the information received at this tertiary hospital—we found this interesting as patients still could not explain what diabetic retinopathy was. The satisfactory services did not, however, seem to affect compliance behaviour.

### 3.3. Underlying Factors: Community-Related

In this section, we outline how support from family and friends, especially adult children and spouses, can motivate patients to attend DR treatments (Table 4). Most compliant patients reported receiving physical (transport and accompaniment) and emotional support from their loved ones. Glucose management improved with the support received from family: patient 8, who was diagnosed with diabetes 30 years prior, stated that his wife also had diabetes, and as such, they motivated each other to follow a healthy diet. Key informant 2 substantiated the value of a supportive family by detailing a case in which a patient failed to manage her diabetes because the family remained ignorant of her health needs and risks.

Key informant 1 suggested that patients default after hearing conspiracy theories in the community about poor ocular treatment outcomes. Despite patients being subjected to both positive and negative theories regarding treatment outcomes, in the current study, this was not cited as a barrier or motivation for them attending their DR treatment.

### 3.4. Individual Factors

We also found multiple individual-related factors to have significant effects on treatment compliance behaviour (Table 5). A poor state of health, whether caused by diabetes complications or other comorbidities, was a critical barrier to diabetic eye care. Two patients in particular, one patient who had a cerebrovascular accident and the other who suffered multiple foot and heart procedures, were unable to attend DR management appointments. One patient was too weak (physically) to comply, and the other too occupied with foot and heart complications. Moreover, the patient who suffered from a diabetic foot ulcer, noted restricted mobility and independence, which further limited compliance behaviour.

Although uncommon among our sample, we found mistrust of health providers, and medication and lifestyle burdens to deter diabetes management compliance. Forgetfulness was another uncommon barrier to compliance.

Key informant 1 mentioned that patients defaulted from DR treatment because they were scared of the procedures. The majority of our patients expressed anxiety over the required eye treatments; however, the anxiety was outweighed by the fear of going blind. Fear of vision loss and concern for ocular health was a critical motivator for treatment compliance behaviour and mitigated treatment-induced fear to some extent.

Diabetes knowledge among patients was limited. All patients knew that diabetes could affect their eyes and vision; however, only one patient could explain how diabetes affects the retina. None of the patients mentioned other ocular complications related to diabetes, such as glaucoma, cataracts, ocular surface disease, or papillopathy. Furthermore, only two patients were aware that they required annual retinal screenings. Both key informants argued that poor health literacy is a barrier to compliance, and they suggested iterative patient education by primary and auxiliary health care providers as a solution.

Patients discussed the financial burden of diabetes management. They explained that eating the prescribed foods can be costly; limited finances force them to eat whatever foods are available—usually a cheap staple such as ‘pap’ (maize-meal)—because they do not have money for a healthier alternative. Interestingly, patients did not mention limited funds as a reason for non-compliance with eyecare appointments at the main NTSS hospital. Furthermore, patients were comfortable with missing a day’s wages in exchange for ocular care, although most were unemployed or pensioners.

### 3.5. SARS-CoV-19-Related Factors

Varying attitudes about SARS-CoV-2, ranging from immense concern to nonchalance, were also reported (Table 6). Despite this, only one patient defaulted on her DR treatment appointments, stating her fear to be infected by SARS-CoV-2. The majority of patients reported being more scared of going blind than contracting SARS-CoV-2.

Contrarily, SARS-CoV-2 was reported to have an immense impact on the rendering of health care services in the NTSS. Patients and key informants reported that many services were scaled-down or suspended altogether during the initial hard-lockdown in South Africa. Patients had limited access to diabetes management services at their primary care facilities during the hard-lockdown period; however, patients reported that chronic-medication delivery services had been implemented. Administrative errors still resulted in one patient being without her diabetes medication for two months. In addition, key informants reported that the health department had deemed primary eye care services non-essential, resulting in the suspension of retinal screening services. This meant patients experiencing vision loss had no clear route to receiving much-needed eye care. Furthermore, all fully compliant patients reported that the main NTSS hospital had cancelled their scheduled DR treatments. This frustrated patients, as many had struggled to receive ophthalmic care appointments and a few patients were already plagued by some form of visual impairment in one or both eyes.

## 4. Discussion

We typified individual, community, and socioeconomic and environmental factors related to compliance behaviour among patients referred for DR treatment. Notable themes included health literacy, state of health, fear of vision loss, accessibility of health services, support from friends and family, and SARS-CoV-2. From our results, we have also established how factors from different levels of the Social Determinants of Health Model interact [14].

All patients came from underserved communities as outlined in Section 2.2. To corroborate the outcomes of Mkhombe [41] and Molapo [42], all patients included in the current study knew that diabetes could affect their eyes. Several patients in our study, however, did not know how diabetes affects ocular health. Mash [43] suggested this could be linked to poor diabetes education in the public sector, resulting from an over-burdened system and severe time constraints faced by health professionals [44]. Indeed, in the current study, we found evidence of this outcome, where both the patients and key informants attested to this by agreeing that the health information imparted in the NTSS may not be always optimal. Previous studies show that high levels of non-compliance with retinal screenings occur when patients do not understand the link between diabetes and its complications [45,46,47]. Hence, there is a need for comprehensive diabetes education and health promotion initiatives in the public sector.

Fear of procedural discomfort and poor visual outcomes after DR treatment has been shown to deter compliance, whereas fear of vision loss resulting from untreated DR motivates patients to comply with treatment [6,48,49,50,51]. However, in the current study we found that, although patients were apprehensive about vision loss or pain from ophthalmological procedures (upon referral for tertiary eye care), they were more afraid of untreated DR that may cause them to lose their vision. Most patients also acknowledged that they would go for DR treatment at the main NTSS hospital during the SARS-CoV-2 lockdown. The fear of going blind as motivation to comply with DR treatment was particularly forthcoming in patients already experiencing vision loss. We concluded that the fear of vision loss mitigated the apprehension of uncomfortable medical procedures and fear of SARS-CoV-2.

Several patients in our study had no visual impairment, while few had some form of visual impairment, ranging from mild to blind. Although no patients mentioned vision loss as a barrier to DR treatment compliance, we can argue that severe visual impairment could deter patients from attending appointments as these individuals would likely require accompaniment which would incur extra financial costs for transportation [6].

We found that poor health had a crippling effect on treatment compliance behaviour. Likewise, previous studies discuss how mobility problems resulting from advanced age and comorbidities can hinder compliance with DR care [52,53]. In cases of poor mobility (common in geriatric and diabetic patients suffering from foot complications), telemedicine and the use of a handheld fundus camera (Optomed Aurora) could be invaluable [54,55]. However, its feasibility in the already over-burdened South African public health system requires testing.

In the current study, we also observed that one patient defaulted from his diabetes medication. In this case, he cited medication-related burden and mistrust of his diagnosis. He also reported that he only took the medication that he perceived as vital (i.e., his heart medication) and defaulted from the rest. He believed that insulin and metformin made him constipated and weakened his vision. Furthermore, he has reverted to drinking sugar-sweetened beverages and believed that he was no longer affected by diabetes. Similar outcomes were reported in other international studies by Choy and Ismail [56], Mohammed, Moles and Chen [57], and Nicolucci et al. [58]. In cases such as these, it becomes critical for health care providers to identify similar patients that may be at risk of feeling over-burdened by medicine intake, so that they may adapt their therapeutic management or implement specific education initiatives accordingly [57]. However, given the time constraints that primary health providers face in the public sector, especially in the South African public health system context, this might prove difficult.

As in similar international studies [6,13,59,60,61], forgetfulness was another barrier to DR treatment compliance behaviour noted in the current research. Chou et al. [62] argue that, in such cases, electronic call-back systems should be evaluated to remind patients of their appointments. However, this would depend on the accuracy of the patient contact information.

Errors in the referral pathways (including the patients’ details) was another important barrier to DR treatment compliance reported by the patients included in the current study. Poor referral systems were evident in the NTSS public health system, where two patients encountered problems with NTSS hospital appointment dates. However, it is important to note that the referral system in this public system has since changed. As motivated by the extensive use of the fourth industrial revolution in the health system, primary health care professionals now communicate with tertiary service providers via a mobile app called Vula Mobile [63]. Vula enables real-time communication between health care providers, reducing the chances of miscommunication. However, an effective communication channel between health services providers and patients still needs to be investigated.

Our findings further suggested that inaccessibility of primary health services and long waiting times were barriers to compliance with annual retinal screenings. International studies [59,64] have also shown that this discourages patients from making appointments, hence they only persevere once they experience vision loss. In the current research, we can link this dilemma to poor diabetes knowledge. This was further associated with an over-burdened public health system and a lack of alternatives for patients from low socioeconomic households.

Contrary to international studies, transportation was not a barrier to compliance for our patients [6,49,62]. As in the Gray and Vawda [65] study, we can ascribe this to our patients reporting that they lived near their day hospitals, with some also reporting having access to various alternative modes of transport. In the current study, several patients reported mainly relying on family and their community members. These outcomes, therefore, highlight the value social support has on mitigating potential barriers to treatment compliance, especially when socioeconomic and other environmental barriers are rife.

Like in the current research, similar South African studies on diabetes management [66,67] outlined that lack of funds hindered patients to procure a healthy diet; a major barrier to successful DR and glucose management. Despite these findings, lack of funds was not a barrier to ophthalmological treatment compliance in patients utilising public health care. This is contrary to international findings [3,59,60] and is assumed to be due to free primary health care (pharmacological interventions and retinal screening services) and partially or fully subsidised tertiary care (DR treatment procedures) in the South African public sector [21].

Furthermore, contrary to other international literature [68,69], difficulty in taking time off of work was not a barrier to compliance behaviour. We may ascribe this to the high level of unemployment and retirement noted among our patients.

Finally, SARS-CoV-2 was a critical barrier to DR treatment in the current study. According to patients and key informants, the main NTSS Hospital cancelled scheduled ophthalmological treatments during the nationwide lockdown, and no patients had received new dates at the time of our interviews. This weighed heavily on patients who had experienced vision loss and were desperate for ophthalmological care. No clear route was available for patients who required urgent specialist intervention. Primary health facilities had also suspended retinal screening services as it was deemed non-essential. In addition, primary facilities only allowed a limited number of patients into the facilities, further limiting access to diabetes management and preventative care. The suspension of vital non-communicable disease management programmes occurred globally [16,70]. If this is not addressed, it could deter patients from seeking care and worsen the backlog in an already over-burdened system, posing further barriers to DR management [15]. However, as this topic is still new, further research on barriers exerted by SARS-CoV-2 and other similar epidemics is needed.

### Limitations

Despite the current study having a lot of strengths, there are limitations that need to be considered when interpreting our findings. First, our study sample was limited to patients who remained attending retinal screenings at their day hospitals. This means that we excluded non-adherent patients who were no longer part of the DR management, whose details were not updated on the database of the organisation performing primary eye care services in the target setting. Missing this cohort may have deprived us of producing more valuable information that could further unpack DR compliance behaviours. However, it is important to highlight that there was no way we could identify these patients without relying on the aforementioned database. We, however, acknowledged the Hipwell et al. [71] findings that suggested barriers to be analogous for compliant and non-compliant patients, which rationalises our exclusion criteria. Moreover, conducting telephonic interviews as opposed to in-person interviews meant that we could not pick up on important non-verbal cues [29]. In this case, at the time there was no way we could breach the lockdown rules and insist on face-to-face interviews. However we acknowledge the work of Novick [72], who argues that patients may be more likely to share sensitive information telephonically. It is also important to note that in the current study we did not seek to quantify the number of patients that falter DR treatment in the NTSS, as well as the level of DR knowledge. Furthermore, we could not specify the causes of visual impairment among patients; as such, it cannot be assumed that visual impairment was as a result of DR. Finally, due to the relative homogeneity and size of the current sample, the generalisability of our findings to the larger South African populations that use public health institutions may not be assumed. Hence, we propose further similar studies and quantitative studies that may be sufficient for generalisability to the larger South African population and health system.

## 5. Conclusions

In the current study, we have demonstrated how socioeconomic and environmental, community, individual, and SARS-CoV-2-related factors affected treatment compliance behaviour in diabetic patients.

Fear of vision loss was a critical individual motivator to comply with DR management, whereas poor health, medication-related burden, forgetfulness, and poor health literacy were notable barriers. Community factors such as social support mitigated potential environmental and socioeconomic barriers such as transport. Furthermore, limited access to DR interventions resulting from over-burdened systems, referral pathway errors, and inaccessible ophthalmology services were critical barriers to receiving timeous care.

Finally, SARS-CoV-2 was an important barrier to DR management as it led to the suspension of primary care services such as retinal screenings, and limited access to diabetes-related medical consultations. In addition, specialist DR treatment procedures at the main NTSS hospital were suspended, with no alternative routes for patients to receive specialist eye care in the public sector.

Because the factors mentioned above are interlinked, this creates a complex problem. Policymakers and managers of similar health care facilities would have to look at initiatives that encompass all levels of determinants of DR treatment compliance behaviours if we are to halt this growing barrier to NCD care in the country. Short-term interventions directed at the individual level and long-term interventions directed at the socioeconomic and environmental, and community levels need to be developed. Immediate action can be taken by improving the implementation of continuous education initiatives targeting newly and previously diagnosed diabetic patients.

## Figures and Tables

**Table 1:** The mean values for select patient demographic information, and best-corrected visual acuities, binocularly and monocularly.

Demographic Factor	Mean or Percentage	
Patients (*n* = 13)		
Age	Mean = 56.4 years ± Standard Deviation = 13.3 years	
Gender:		
Female	61.5%	
Male	38.5	
Diabetes mellitus type:		
Type 1	7.7%	
Type 2	84.3%	
Unconfirmed	7.7%	
Diabetes mellitus duration:		
≤4 years	7.7%	
>4 years	84.6%	
Unconfirmed	7.7%	
Diabetes mellitus treatment:		
Metformin	30.8%	
Insulin	46.2%	
Metformin and insulin	15.4%	
Unconfirmed	7.7%	
Visual acuity (VA) with correction at distance:	Binocular (*n* = 13)	Monocular (*n* = 26)
6/6 ≤ VA < 6/12	69.2%	57.7%
6/12 ≤ VA ≤ 6/18	15.4%	19.2%
VA < 3/60	7.7%	15.4%
Unavailable	7.7%	7.7%

**Table 2:** Classification of compliance.

Classification	Definition	Patients	
		Sample Size (*n*)	Percentage (%)
Fully compliant	Being present for all NTSS hospital DR treatment appointments (excluding instances where appointments were cancelled by the NTSS hospital due to the COVID-19 pandemic)	8	61.5
Partially compliant	Being present for the initial DR treatment consultation at the NTSS hospital, but missing any of the follow-up appointments without rescheduling	1	7.7
Not compliant	Did not attend any NTSS hospital DR treatment or consultation appointments	4	30.8

**Table 3:** Socioeconomic and environmental factors.

Theme	Quotes
Accessibility of health services	“*…it’s easy because I don’t live far from it (day hospital)*” (Patient 10).
	“*…we just ask the pastor’s wife to take me, because I can’t walk*” (Patient 5).
	“*I take a taxi to Bellville, and from Bellville to the (NTSS) hospital.*” (Patient 12).
	“*Look, to get your retinal screening on a list at the day hospital (meaning to get an appointment-name on the list) is almost like… getting a rock out of water.*” (Patient 8).
	“*… they just said I must go to (the main NTSS) hospital… my appointment was without a referral letter, they said the letter is at (the NTSS) hospital... when I got there, they told me that my name isn’t anywhere on the list.…*” (Patient 10).
	“*…I have the (NTSS) hospital’s number, but when you phone them then they put you through to... that clinic (eye clinic) and you wait and the music plays in your ears and at the end of the day your airtime is finished*” (Patient 13).
Quality of health services	“*My experience with the diabetes and the primary eyecare providers… they didn’t provide a lot of info...*” (Patient 1 carer).
	“*…It depends patient by patient… I am able to do as much as I would like to do […] With time constraints and working conditions, and the rooms we get, the time we have with the people, just are not naturally conducive to testing… we might not have more time to spend on what we need to spend…*” (Key informant2).
	“*They told me what I needed to know, just like the basics of what I needed to know and just rushed through the next patient*” (Patient 9).
	*I’m very impressed with the treatment (at the NTSS Hospital) … it’s quite long. I mean, I was there the morning… I got out of there at 4 in the afternoon, but … there are so many people… they do so many different tests, and everything takes long, I mean that is understandable. For me it was the best […] At every person (testing station) they explained*” (Patient 7).

**Table 4:** Community factors.

Theme	Quotes
Support from family and friends	“*I tell my children at home, they care… the oldest one (daughter), she’s very… concerned… she said she thinks it’s a good idea, so I can take care of my health, because I am also looking after the little ones (grandchildren) at home*” (Patient 13).
	“*My wife and I are both diabetic… we eat our food that we buy… (It’s) A bit expensive but… You can’t not […] there are two in my family that are also diabetic… they eat and do as they please.*” (Patient 8).
	“*There are some who, who have the support structure and those are the ones who never have problems in general. But the majority is people who don’t have a support structure and as a result don’t comply*” (Key informant 2).
Conspiracy theories about treatment outcomes	“*But then you do get those that don’t want to go because they hear stories from other people […] maybe other people told them that they went and now they see worse than they did before…*” (Key informant 1).
	“*…she got to [the NTSS] hospital. They did her eyes. But she says she’s so satisfied, she’s back at work so satisfied, she can drive again and all of that*” (Patient 8).
	“*…my wife was sceptical at the time… when the doctor said I required laser treatment, and when the appointment was made, that’s when my wife started getting a bit sceptical… because the people hear things from other people…*” (Patient 4).

**Table 5:** Individual factors.

Theme	Quote
State of health	“*…her grandson was here at the time and I discussed it with the family and they said no rather not because they don’t want anyone poking around because* P1 *was quite sick at the time and they felt that she would not be able to handle much more*” (Patient 1 carer).
	“*…because I was in the hospitals for surgeries and other things, I never got around to it (eye examination) […] I struggled with my foot to heal. I was bedridden for months…*” (Patient 4).
Medication-related burden	“*Then eventually, to tell you the truth, I got fed up with having to struggle with the pills […] I decided to look at the pills that are important to me, in my life […] then I decided, over a period of time, that I’m tired of the Metformin I’m drinking, because it makes me very constipated and so on. And then I left it and I’ve been off it for over two months now. And the same with the Insulin, I don’t even use Insulin anymore […] then I just wondered if the pills were even worth taking? […] I drink cooldrink and all of those things. I’m living normally, like how I used to live […] I wondered whether the diabetes medication didn’t maybe have a negative impact on my life, because my eyes weakened so much.*” (Patient 4).
Forgetfulness	“*…And then I did go to the (NTSS) hospital. In May, June, July I was supposed to go for the injections in the eye. Then this month they would have checked to see, and then they would’ve maybe done the laser… But because I couldn’t find my day hospital card and didn’t know what dates I needed to go, I didn’t attend those three months…*” (Patient 13).
Fear of vision loss	“*The main thing, I think they (patients) are scared*” (Key informant 1).
	“*I was scared. I didn’t know what to expect*” (Patient 8).
	“*…if I don’t do it then I’ll eventually go blind*” (Patient 3); “*…probably my fear that I’ll go blind if I don’t go*” (Patient 7).
Health literacy	“*…so diabetes can affect your eyes, your eye health, uhm... can cause blurry vision… In severe cases it could cause blindness… that’s the extent I know about it*” (Patient 9).
	“*So it’s like they say, the sugar, it affects the veins in your eyes […] they explain to you*” (Patient 3).
	“*…they never said we must go for an eye test (annually)… They won’t tell you that you have to go for an eye test now*” (Patient 10).
	“*[…] as soon as they become diabetic, they should know the consequences. So, the main thing is patient education should be increased*” (Key informant 1).
Financial trade-offs	“*…to eat healthy, for a diabetic, is expensive… if the money isn’t there… and you only have pap and bread and not even vegetables really... I mean households are struggling (financially)*” (Patient 3).
	“*…look, you lose a day’s wages, but you don’t care, it’s for your own health. I will never skip an appointment. Even if I have to lose a day’s wages*” (Patient 6).
	“*I’m not working at the moment. They retrenched me 10 years ago already*” (Patient 12).

**Table 6:** SARS-CoV-2-related factors.

Theme	Quotes
SARS-CoV-2	“*We have to be very careful of COVID (SARS-CoV-19), so the people are standing outside by the gates. Even if you phone ahead and say you are coming in, you have to stand outside…*” (Patient 1 carer).
	“*I didn’t attend the (NTSS hospital DR) treatment appointment because of COVID… I was just worried because I am a chronic patient, and now the COVID is so terrible and it will affect me*” (Patient 2).
	“…*I’m not afraid of COVID*” (Patient 8).
	“*…due to the pandemic we’ve had to suspend our services completely because the clinics needed to reduce the number of patients visiting the clinic. So, we have not been running our services, or our patients have not been getting retinal screenings or eye screenings for the past, how many months…*” (Key informant 1).
	“*No, at the moment the public hospitals are only dealing with eh, emergencies when it comes to eyecare, so unless you are losing vision with an accident, they do not see routine cases…So there is no way to get help at the moment*” (Key informant 2).
	“*I was supposed to go to (the NTSS) hospital recently, for my eyes, because they would have done laser. But then the appointments got cancelled due to the COVID story […] I know they cancelled all the (NTSS hospital ophthalmology) appointments when the COVID-19 came around... And I can understand that they are worried about the high-risk people, but I am worried about my eyes […] To get an appointment at the eye specialist there is difficult because there is such a long waiting list. And when I eventually got an appointment there, it was like a breakthrough, and then the COVID-story happened and I’m just wondering if I’ll get another chance*” (Patient 4).

## Data Availability

The data presented in this study are available on request from the corresponding author and with permission from the Biomedical Research Ethics Committee of the University of the Western Cape and the Western Cape Provincial Research Council.

## Supplementary Materials

The following are available online at https://www.mdpi.com/article/10.3390/ijerph182212209/s1, Text S1: Semi-structured interview guide—patients; Text S2: Semi-structured interview guide—key informants.

## Abbreviations

DR	Diabetic retinopathy
NCD	Non-communicable disease
NTSS	Northern/Tygerberg sub-Structure
SARS-CoV-2	Severe acute respiratory syndrome coronavirus 2
