# Supplementary material for: Exploring Factors Associated with Diabetic Retinopathy Treatment Compliance Behaviour in Cape Town, South Africa
Source: Int J Environ Res Public Health. 2021 Nov 20;18(22):12209. doi: 10.3390/ijerph182212209 (PMC8617604; doi:10.3390/ijerph182212209)
Supplement: Supplementary file 1 [file ijerph-18-12209-s001.zip › S1 Text_ Semi-structured interview guide _ patients.pdf]

S1 Text: Semi-structured interview guide - patients

1. Can you tell me about the day hospital that you visit for treatment and information (for diabetes)?
   Prompts
   - Is it easily accessible?
   - How do you feel about the services?
   - How do you feel about the staff?
   - How do you feel about the information they give you?
2. How long have been living with diabetes?
3. Can you tell me what you know about diabetes and eye health?
   Prompts
   - Can you elaborate?
   - Where did you get this information from?
   - What information has the day hospital provided regarding eyecare for diabetic patients?
   - Do you ever get your information from friends, family or the internet (google)?
4. Are you aware that you require an eye examination every year due to your diabetic condition?
5. Can you tell me what you were told about diabetic retinopathy treatment?
   Prompts
   - Who told you?
6. Is there anything related to diabetic retinopathy you wish medical staff explained better?
7. Did the person who referred you for treatment tell you what to expect when going for treatment?
8. Can you tell me about finding out that you have diabetic retinopathy?
   Prompts
   - Who informed you?
   - What did they say?
   - Can you explain how you felt?
   - Why?
9. Did you attend the eye treatment?
10. Was it easy for you to get to Tygerberg Hospital?
    - Did you have to rely on someone for transport?
    - Was the distance to travel or cost of travelling a problem?
11. Did you have to take time off of work?
12. Can you tell me about the experience you had when you went for diabetic retinopathy treatment?
    Prompts
    - Was the procedure comfortable or uncomfortable?
    - Did anyone explain to you what they were doing and why?
    - Would you go for another treatment if it was required?
13. Can you tell me about some times when you missed your treatment appointments?
14. What made it difficult for you to attend appointments?
    Prompts
    - What was going on in your life?
    - Did you tell friends and family about needing the treatment?
    - Were your family and friends supportive?
    - What were your experiences with Tygerberg Hospital and its staff members?
15. What were the main factors that motivated you to attend appointments?
16. Has it been easier or more difficult to get the required diabetic retinopathy treatment since the coronavirus lockdown?
    Prompts
    - Can you explain what process you went through?
17. Did you forgo eye appointments due to corona-virus concerns?
18. Has the coronavirus pandemic stopped you from seeking medical care for diabetic retinopathy?
    Prompts
    - In what way?
    - Are you worried about going to a hospital during this time?
    - Why are you worried?
    - Were your treatment or follow-up dates changed as a result of COVID-19 related problems?
