# Supplementary material for: Exploring Factors Associated with Diabetic Retinopathy Treatment Compliance Behaviour in Cape Town, South Africa
Source: Int J Environ Res Public Health. 2021 Nov 20;18(22):12209. doi: 10.3390/ijerph182212209 (PMC8617604; doi:10.3390/ijerph182212209)
Supplement: Supplementary file 1 [file ijerph-18-12209-s001.zip › S2 Text_ Semi-structured interview guide _ key informants.pdf]

S2 Text: Semi-structured interview guide - key informants

1. Please describe your role within the Retinal Screening Programme
   Prompts
   - Can you elaborate on your duties relating to the retinal screenings?
   - Are there more people in your team?
2. What information regarding diabetic retinopathy do you provide to diabetic patients during their retinal screening?
   Prompts:
   - Do the patients seem well informed regarding diabetic retinopathy?
   - Are you aware of any other medical professionals that inform them of diabetic retinopathy?
   - Do you think the day hospital provides enough information regarding diabetic retinopathy?
3. Do the other members of your team offer patients information regarding diabetic retinopathy?
   Prompts:
   - What information do they offer patients?
   - How is it different from the information you offer patients?
4. Do you know if the day hospital provides them with any info?
   - Do you think it would help if health promoters take on some of those education responsibilities?
5. What information regarding the **treatment** of diabetic retinopathy is provided to diabetic patients when they are referred?
   Prompts:
   - Are patients informed of the available treatments?
   - Are patients informed about what they can expect regarding treatment?
6. How do diabetic patients react when you inform them that they need to be referred for diabetic retinopathy and require treatment?
7. What support is provided by the Retinal Screening Programme/your NGO for patients who need treatment?
8. What are some of the challenges you have faced when referring participants for diabetic retinopathy treatment?
   Prompts
   - Were there challenges relating to the patient/ patient compliance?
   - Were the challenges related to the referral protocol with the tertiary/treating institution?
   - What were the challenges related to the diabetic retinopathy treatment facility or its staff members?
9. How would you address challenges brought up in the previous question?
10. Have any patients informed you that they have missed their scheduled treatment appointments?
    Prompts:
    - Did they give a reason?
    - What were the reasons?
    - To your knowledge, did they receive a new date for treatment?
    - Was it relatively easy for them to get another date?
11. What, in your opinion, are the main barriers to compliance among your patients?
12. How does the health care facility (day hospital AND Tertiary Hospital sides) help address these problems?
13. What steps/implementations/measures do you think can be taken by day hospitals or your NGO/retinal screening programme to reduce the non-compliance rates regarding DR treatment?
14. Has the coronavirus pandemic affected your ability to perform retinal screenings and refer patients for diabetic retinopathy treatment?
    Prompts
    - How has it affected your ability to perform at work?
    - How do you feel about this?
15. If you are unable to provide the retinal screening services during this time, are you aware of any other routes that patients within the public health system can take to access primary eyecare services and retinal screenings?
    Prompts
    - Can you describe the routes?

- If there aren't any other routes, what do you think will happen with patients who urgently require diabetic retinopathy treatment?
- Do you think urgent cases will receive timeous treatment?
